# Beyond Fluid Therapy: The Role of Vitamin C, Steroids, and Thiamine in Sepsis Management

**DOI:** 10.7759/cureus.84666

**Published:** 2025-05-23

**Authors:** Courage O Idahor, Olamide Ogunfuwa, Ndidiamaka Ogbonna, Augustina Adigwe, Omo A Ogbeide

**Affiliations:** 1 Emergency Medicine, Nottingham University Hospitals NHS Trust, Nottingham, GBR; 2 Emergency Medicine, Sandwell and West Birmingham NHS Trust, Birmingham, GBR; 3 Family Medicine, Edo State University Iyamho, Uzairue, NGA; 4 General Medicine, Zaporizhzhia State Medical University, Zaporizhzhia, UKR; 5 General Practice, NES Healthcare, Buckinghamshire, GBR; 6 General Practice, Chaucer Hospital, Canterbury, GBR

**Keywords:** biomarkers, corticosteroids in sepsis, inflammation, metabolic resuscitation, mitochondria, oxidative stress, sepsis, septic shock, thiamine supplementation in sepsis, vitamin c therapy

## Abstract

Sepsis remains a major contributor to mortality among critically ill patients, with sepsis-induced metabolic dysfunction significantly worsening outcomes. As metabolic dysfunction plays a key role in the pathogenesis of sepsis, recent interest has grown around metabolic resuscitation therapies as potential adjuncts to traditional fluid resuscitation strategies. This narrative review evaluates current evidence regarding the role of vitamin C, thiamine, and corticosteroids in improving sepsis outcomes. Early studies suggested that vitamin C may reduce organ dysfunction and vasopressor requirements; however, more recent randomized trials have produced inconsistent results, with some findings even indicating potential harm in certain patient groups. Similarly, the use of corticosteroids in sepsis management has shown mixed outcomes. Thiamine has demonstrated possible renal protective effects and improved lactate clearance, although its impact on mortality and vasopressor needs remains inconclusive. Combination therapy with hydrocortisone, vitamin C, and thiamine (the HAT protocol) has been associated with reduced vasopressor duration but has not consistently improved survival or other major clinical endpoints, despite its apparent safety. Overall, while vitamin C, corticosteroids, and thiamine present a theoretically attractive strategy in sepsis management, clinical results remain debated. Corticosteroids currently have the strongest supporting evidence for use in septic shock, while vitamin C and thiamine remain investigational therapies and are not recommended for routine use outside clinical trials. Future research should explore biomarker-guided, precision-medicine approaches to better identify patients who might benefit most from metabolic resuscitation, and large-scale randomized controlled trials are needed to clarify optimal timing and dosing strategies.

## Introduction and background

Sepsis occurs when the body's response to an infection becomes dysregulated, resulting in potentially life-threatening organ dysfunction due to widespread systemic inflammation, immune system imbalance, and microvascular abnormalities. It results from the failure of the body’s immune and physiological mechanisms to maintain homeostasis in response to an infectious insult, ultimately contributing to multi-organ impairment and increased mortality risk if not promptly recognized and treated [1-3]. Timely and appropriate hemodynamic resuscitation is essential for enhancing survival rates in patients with septic shock, as the effectiveness of resuscitation directly influences morbidity and mortality rates [4-6]. However, even when resuscitation goals are met, a considerable number of septic patients develop multiple organ dysfunction syndrome (MODS) and ultimately succumb to the illness, indicating that additional factors contribute to the syndrome’s pathophysiology [4,7,8]. The occurrence of sepsis and septic shock differs across continents and countries, highlighting their significance as global health challenges [4,9]. Worldwide, sepsis has an age-standardized incidence rate of 677.5 cases per 100,000 individuals. Sub-Saharan Africa, Oceania, and South Asia have reported the highest rates [4,9]. Significant advancements have been made in recent years in the area of hemodynamic optimization for sepsis and septic shock. Current recommendations emphasize early and aggressive, yet controlled, fluid resuscitation, along with the use of albumin in fluid management, inotropic therapy, and extracorporeal life support for refractory cases [4,10-15]. Concurrently, improvements in hemodynamic monitoring have facilitated more precise therapeutic adjustments, thereby reducing the risks associated with both over- and under-treatment. The integration of non-invasive methods for monitoring cardiac output, including echocardiography and the ultrasonic cardiac output monitor (USCOM), is becoming increasingly widespread in intensive care units (ICUs) globally [4,16,17]. Additionally, key biomarkers, including central venous oxygen saturation (ScvO₂), clearance of lactate and the arterial-venous CO₂ gradient have been instrumental in informing treatment choices and enhancing patient outcomes [4,18,19]. Despite these significant advancements, sepsis-related mortality rates remain persistently high [4].

The pathophysiology of sepsis is intricate, characterized by an imbalance in inflammatory responses and dysfunction of the immune system, neuroendocrine-immune network disturbances, mitochondrial injury, endothelial dysfunction, coagulation abnormalities, and impaired autophagy [20-23]. This inflammatory response may progress to immunosuppression, disrupting key metabolic pathways such as glycolysis, fatty acid oxidation, and oxidative phosphorylation, with severity influenced by genetics, comorbidities, and pathogen type [20,22]. These interconnected processes ultimately contribute to organ dysfunction and increased mortality risk [20,21]. Invasive pathogens such as bacteria, fungi, parasites, and viruses trigger activation of the innate immune system, including macrophages, monocytes, neutrophils, and natural killer cells [20-22]. Pathogen-associated molecular patterns (PAMPs) and damage-associated molecular patterns (DAMPs) are recognized and bound by pattern-recognition receptors (PRRs) [20,21,24]. Both PAMP and DAMP interactions trigger the activation of neutrophils and specific signaling pathways that regulate inflammation, adaptive immune responses, and cellular metabolism. This series of events results in the generation of inflammatory mediators, which facilitate the emergence of pro-inflammatory, pro-apoptotic, pro-adhesive, and pro-coagulant phenotypes [20,21,25]. The process of inflammation entails the synchronized actions of leukocytes, cytokines, oxygen radicals, endothelial cells, along with the complement and coagulation systems. While these components are fundamental to innate immunity, the disruption of these factors significantly contributes to the onset of sepsis [20,26]. The initial inflammatory response during sepsis also plays a major role in the onset of oxidative stress [20,27]. This happens when the generation of reactive oxygen species (ROS) exceeds the body's ability to neutralize them with its antioxidant defenses, resulting in an accumulation of oxidants [20,28,29]. Notably, the generation of ROS acts as a driver, in part by activating the NF-κB signaling pathway, which is crucial for inflammation and a consequence of inflammation, further amplifying the pathological process [20,27,29].

ROS are essential for maintaining normal cellular function and play a pivotal role in various cell signaling pathways. However, when oxidative stress occurs, the overproduction of ROS overwhelms the body’s antioxidant defense mechanisms. This results in detrimental interactions with essential cellular components such as proteins, carbohydrates, nucleic acids, and unsaturated lipids. As a result, these interactions contribute to structural and functional damage within the cell, ultimately disrupting homeostasis and potentially leading to cell dysfunction or death [20,28,30]. The mitochondrial electron transport chain is a key source of ROS. In sepsis, mitochondria undergo structural changes, including proton leakage across the inner membrane, increased membrane permeability, and potential loss of function. As mitochondrial dysfunction worsens, it generates more ROS and releases pro-apoptotic proteins, triggering cell death. This disruption also impairs adenosine triphosphate (ATP) production, leading to an energy crisis at the cellular level, which has been linked to higher mortality in sepsis patients [20,27-31]. Inflammation and oxidative stress significantly contribute to endothelial dysfunction, which is a primary characteristic of sepsis that leads to organ damage [20,23,25,26]. The breakdown of the endothelial glycocalyx, which serves as a protective barrier to uphold vascular integrity, is a significant result of sepsis-related endothelial dysfunction. Composed of proteoglycans, glycoproteins, glycolipids, glycosaminoglycans, and plasma proteins, the glycocalyx prevents excessive immune cell adhesion. Its degradation weakens the vessel barrier, allowing immune cells to attach to the endothelium, which worsens inflammation [20,32]. Furthermore, increased production of nitric oxide (NO), the body’s primary vasodilator, further disrupts endothelial function by increasing permeability and promoting platelet adhesion. These changes contribute to acute endothelial dysfunction, leading to hypotension, impaired oxygen delivery, and organ ischemia. The endothelium also plays a key role in anticoagulation, and its dysfunction triggers the coagulation cascade, increasing the risk of sepsis-associated coagulopathy [20,23,32,33]. Overall, the intricate interaction among inflammation, oxidative stress, endothelial dysfunction, and mitochondrial impairment propels the advancement of sepsis. These processes disrupt cellular and organ function, ultimately leading to multiple organ dysfunction, which greatly influences the severity and outcomes of sepsis [20].

The previous discussion clearly indicates that mitochondrial dysfunction plays a crucial role in the development of sepsis and the advancement to multiple organ dysfunction syndrome. Despite significant advancements in sepsis management, mortality rates remain unacceptably high [4]. As a result, focusing on mitochondrial function has become a promising therapeutic strategy to prevent, alleviate, or even reverse MODS and improve survival in sepsis. This strategy, often referred to as “metabolic resuscitation,” aims to restore cellular energy balance and mitigate the detrimental effects of mitochondrial impairment [4]. A crucial element of metabolic resuscitation is thiamine (vitamin B1), which is a water-soluble vitamin vital for cellular energy metabolism. In patients with septic shock, both relative and absolute thiamine deficiency can occur, further contributing to mitochondrial dysfunction and metabolic failure [4]. One of the significant consequences of thiamine deficiency in sepsis is lactic acidosis, a marker of disease severity that has been associated with worse outcomes. Thiamine supplementation has been shown to help reduce lactate levels in sepsis, making it a potentially valuable intervention, particularly in patients at high risk of deficiency. These include individuals with malnutrition, chronic alcoholism, hyperemesis gravidarum, chronic wasting diseases, renal replacement therapy, anorexia nervosa, gastric bypass surgery, and refeeding syndrome. Given its role in cellular metabolism and organ function, thiamine replacement may serve as a simple yet effective strategy in the broader effort to mitigate sepsis-induced organ dysfunction [4,34].

Emerging evidence suggests that supplementation with specific micronutrients may help mitigate sepsis-induced mitochondrial dysfunction by targeting oxidative stress and cellular energy failure. Vitamin C, vitamin E, selenium, and zinc have demonstrated antioxidant and cytoprotective effects in experimental studies. For instance, vitamin C has been shown to enhance the expression of antioxidant enzymes, while vitamin E and selenium protect mitochondria and tissues from oxidative damage. Zinc contributes to the reduction of oxidative stress and the modulation of inflammatory processes [4,35,36]. Beyond these micronutrients, other potential interventions, such as coenzyme Q, L-carnitine, cytochrome oxidase (CytOx), and melatonin, have been proposed to support mitochondrial function in sepsis. However, while preclinical data are promising, the clinical efficacy of these strategies remains uncertain and requires further investigation [4,37-42]. This narrative review aims to evaluate the evidence supporting metabolic resuscitation and discuss future research directions.

## Review

Pathophysiological basis for metabolic resuscitation in sepsis

Oxidative Stress and Endothelial Dysfunction

Oxidative stress plays a central role in sepsis pathogenesis, contributing to cellular damage, inflammation, and impaired mitochondrial function [20,30]. Although ROS play a crucial role in normal cellular signaling, the overproduction of ROS during sepsis results in the oxidation of proteins, lipids, and nucleic acids, driving tissue injury and organ dysfunction. Key ROS implicated in sepsis include superoxide (O_2_^•−^), hydrogen peroxide (H_2_O_2_), peroxynitrite (ONOO^−^), hydroxyl radicals (^•^OH), and hypochlorous acid (HOCl) [20,28]. A major consequence of excessive ROS production is the activation of the NF-κB pathway, which enhances inflammation by triggering the release of pro-inflammatory cytokines, including IL-1β, IL-6, and tumor necrosis factor-alpha (TNF-α) [20,27,29]. Additionally, ROS promote leukocyte recruitment and further cytokine synthesis. This leads to a continuous cycle of inflammation and oxidative stress [20,27]. The antioxidant defense system comprises enzymes such as superoxide dismutase (SOD), glutathione peroxidase (GSH-Px), and catalase (CAT), and is responsible for neutralizing excess ROS [20,43]. However, sepsis disrupts this balance, as seen in experimental models where hepatic CAT and GSH-Px activity decreases while SOD levels rise, leading to a dysfunctional antioxidant response [20,44]. Further compounding oxidative stress, sepsis upregulates ROS-producing enzymes, including NADPH oxidases (NOX1-5, Duox1-2) and nitric oxide synthase (NOS) [20,28]. NOS enzymes use NADPH to generate superoxide, which fuels further ROS production, while inducible NOS (iNOS) is overexpressed during sepsis, which results in the overproduction of nitric oxide. NO interacts with superoxide to produce the highly reactive peroxynitrite, exacerbating oxidative injury and mitochondrial dysfunction [20,28,30,45]. The mitochondrial electron transport chain serves as a significant source of ROS during sepsis [20,28,29]. Mitochondrial dysfunction, characterized by membrane permeability transition, proton leak, and impaired ATP synthesis, further enhances the production of ROS and triggers the release of pro-apoptotic proteins, which facilitates cell death. This disruption in ATP generation contributes to cellular energy failure, a critical factor in multiple organ dysfunction syndrome [20,28,29,31]. Moreover, sepsis-induced mitochondrial dysfunction leads to cytopathic hypoxia, where tissues are unable to effectively utilize oxygen despite normal arterial oxygen levels [20,29,31,45]. In response to hypoxia, sepsis triggers the activation of hypoxia-inducible factors (HIFs; HIF-1, HIF-2), which regulate genes involved in coagulation and vascular homeostasis. These alterations contribute to endothelial dysfunction, impaired oxygen delivery, and metabolic failure, further worsening organ injury [20,46].

Endothelial dysfunction is a key factor in the development of sepsis, leading to compromised blood flow, organ hypoxia, and systemic inflammation. Under normal conditions, the vascular endothelium regulates blood flow, prevents excessive coagulation, and maintains barrier integrity. However, in sepsis, a combination of inflammation and oxidative stress damages the endothelium, triggering a cascade of pathological events that worsen organ dysfunction and mortality [20,23,25,26,47]. Pathogens trigger the activation of endothelial cells via pattern recognition receptors (PRRs). This triggers the activation of inflammatory pathways, including NF-κB and MAPK [20,33]. Cytokines and adhesion molecules such as E-selectin, ICAM-1, and VCAM-1 are produced as a result, facilitating the adhesion and migration of leukocytes, further fueling local inflammation [20,23,32]. Additionally, the disruption of the endothelial glycocalyx, a critical barrier that regulates vascular permeability, exposes the endothelium to immune cell adhesion and inflammatory damage [20,32]. Increased endothelial permeability, driven in part by excessive nitric oxide production, contributes to hypotension, tissue hypoxia, and organ failure [20,23,32,33]. Beyond inflammation, endothelial dysfunction also plays a key role in sepsis-associated coagulopathy. The endothelium loses its anticoagulant properties, leading to dysregulation of clotting factors such as thrombomodulin, antithrombin, and tissue factor. This shift toward a procoagulant state results in widespread micro-thrombosis, exacerbating hypoxia and ischemic damage. Notably, thrombocytopenia, often observed in sepsis, correlates with disease severity and poor outcomes due to ongoing platelet activation and fibrin clot formation. Given the pivotal role of oxidative stress in sepsis pathophysiology, metabolic resuscitation strategies aim to restore redox balance and mitochondrial function. Antioxidant-based interventions, including thiamine, vitamin C, vitamin E, selenium, and zinc, have shown potential in experimental models by mitigating, oxidative damage and supporting mitochondrial integrity. However, further clinical studies are needed to establish their efficacy in improving outcomes in sepsis patients [4].

Mitochondrial Dysfunction and Bioenergetic Failure

Mitochondrial dysfunction is a key part of the pathophysiology of sepsis and plays a major role in the development of organ failure through energy collapse at the cellular level. During sepsis, mitochondria in immune cells and various tissues come under intense stress. This leads to structural distortion, loss of mitochondrial content, and a marked drop in respiratory activity, all of which contribute to reduced ATP production and energy failure [48]. Several processes contribute to mitochondrial damage in sepsis. These include the build-up of reactive oxygen and nitrogen species (ROS and RNS), faulty mitophagy, disrupted mitochondrial dynamics, and increased permeability of the mitochondrial membrane [49]. One major issue is the excessive production of nitric oxide, which occurs both in the cytoplasm and within the mitochondria through iNOS [48,49]. This NO can be converted into other reactive nitrogen species like nitrite and nitrogen dioxide. At the same time, ROS are generated in large amounts by NOX enzymes and the mitochondrial electron transport chain. When nitric oxide interacts with superoxide, it generates peroxynitrite, which is a highly toxic substance. These reactive molecules lead to extensive damage to mitochondrial DNA, lipids, and proteins, which in turn further disrupts mitochondrial respiration and structure [48]. Mitophagy, the process by which damaged mitochondria are selectively broken down, becomes impaired during sepsis [48,50]. This allows dysfunctional mitochondria to accumulate [48,51]. The best-understood pathway regulating mitophagy is the PINK1-Parkin pathway. When the membrane potential of the mitochondria diminishes, PINK1 accumulates on the outer mitochondrial membrane and attracts Parkin. Parkin then labels proteins on the damaged mitochondria for degradation. If this pathway does not work properly, damaged mitochondria remain in the cell, continue generating ROS, and worsen cellular stress [48]. Mitochondrial dynamics, which refer to the balance between fusion and fission, are also affected. Fusion helps maintain mitochondrial health by promoting the exchange of genetic and protein content, while fission isolates damaged sections of mitochondria [48,52,53]. In sepsis, this balance is lost, and excessive fission takes over. As a result, mitochondria become fragmented and less efficient. Research indicates that minimizing excessive fission may aid in restoring mitochondrial function and enhancing energy production [48,54]. Another factor is mitochondrial membrane permeabilization. This results in mitochondrial swelling, loss of internal contents, and disruption of the membrane potential, which is crucial for ATP synthesis. This occurs through several mechanisms, including mitochondrial outer membrane permeabilization (MOMP), mitochondrial permeability transition (MPT), and a newer process involving gasdermins (GSDMs). These changes contribute to energy failure and play a part in triggering cell death [48,55]. Collectively, these mechanisms lead to substantial mitochondrial impairment and a decrease in energy production, even when oxygen delivery to tissues is not compromised. This bioenergetic failure is now seen as a major factor in the progression of organ failure in septic patients [48].

Mitochondria-targeted therapies in sepsis: Developing effective treatment options for patients with sepsis remains a pressing need, especially for those in critical condition. Interestingly, studies have shown that individuals who survive sepsis often mount an early response by boosting mitochondrial biogenesis and antioxidant defenses. This suggests that preserving mitochondrial stability and function might be a promising approach in managing sepsis [48,56]. Because reactive oxygen species are recognized for their ability to induce mitochondrial damage and promote inflammation, antioxidant-based treatments have drawn considerable interest. A number of mitochondria-targeted antioxidants have been developed and studied, with MitoQ, MitoVitE, and MitoTempol among the most widely explored [48,57]. MitoQ, although not yet approved by the US FDA, is a compound that links ubiquinone (a form of coenzyme Q10) to a lipophilic molecule called triphenylphosphonium (TPP^+^), allowing it to specifically accumulate within mitochondria [48,58]. Research suggests that MitoQ can help protect mitochondrial integrity during sepsis, reduce the release of inflammatory cytokines, and improve liver and kidney function [48,59]. MitoVitE is a form of vitamin E designed to target mitochondria, linked to TPP^+^, and has demonstrated comparable advantages. It supports mitochondrial function, helps reduce inflammation, and limits organ damage [48,58]. MitoTempol, another targeted antioxidant, is made by combining Tempol (a nitroxide compound with antioxidant properties) with TPP^+^. In animal studies, MitoTempol has been shown to reduce levels of the inflammatory marker IL-1β, lower oxidative stress in the kidneys, and improve kidney function in the context of sepsis [48,60,61]. In addition to antioxidants, a deficiency in NAD+, a crucial molecule for energy metabolism and the regulation of oxidative stress, has been noted in cases of sepsis. Consequently, there has been an increasing interest in NAD+ supplementation as a potential therapeutic approach [48,62,63]. Several other compounds, which do not mainly serve as antioxidants, are currently under investigation for their potential to support mitochondrial health. These include agents like Urolithin A, Mdivi-1, Cyclosporin A, and Metformin. Each of these targets different aspects of mitochondrial function, such as enhancing mitophagy, stabilizing mitochondrial dynamics, and preventing membrane damage. Early research suggests that these agents may help improve outcomes by limiting mitochondrial injury and maintaining energy balance during sepsis [48].

Immunosuppression and Dysregulated Inflammatory Response

Sepsis is marked by an uncontrolled immune reaction to infection, usually starting with a phase of overwhelming inflammation followed by a state of immune suppression or “immune paralysis” [64]. This shift plays a crucial role in disease progression and often determines patient outcomes. In the early stages, the immune system releases a surge of pro-inflammatory cytokines like TNF-α and IL-6 that play a role in controlling the infection; however, their excessive production can lead to tissue damage and organ dysfunction [65]. As sepsis advances, the immune system begins to downregulate its response. Anti-inflammatory cytokines like IL-10 become dominant, aiming to curb the inflammation. However, this compensatory response can overshoot, leading to profound immunosuppression [66]. This immune paralysis is marked by lymphocyte apoptosis, reduced antigen presentation, and impaired immune cell function. As a result, patients become more vulnerable to secondary infections and may fail to clear the primary infection effectively [64]. The balance and timing of these cytokine shifts are critical and continue to be a major focus in understanding sepsis pathophysiology and developing targeted therapeutic approaches.

Role of vitamin C in sepsis management

Vitamin C has garnered interest as a possible supplementary treatment for sepsis because of its antioxidant, anti-inflammatory, and immunomodulatory properties. It plays a critical role in endothelial function, catecholamine synthesis, and neutralization of reactive oxygen species, all of which are disrupted in sepsis [67].

Key Clinical Studies

Early interest was sparked by observational and small-scale interventional studies suggesting improved outcomes with high-dose intravenous vitamin C, particularly when used as part of a combination therapy (e.g. the “Marik protocol”) involving hydrocortisone and thiamine [68]. These studies reported reductions in organ dysfunction, vasopressor requirements, and even mortality. However, more recent large-scale randomized controlled trials (RCTs) have produced conflicting results. The Vitamin C, Hydrocortisone and Thiamine in Patients With Septic Shock (VITAMINS) trial, for example, showed no notable difference in the primary outcome of time alive and free from vasopressors over a seven-day period between patients treated with combination therapy and those who received only hydrocortisone [69]. Similarly, the Lessening Organ Dysfunction with Vitamin C (LOVIT) trial reported that high-dose vitamin C monotherapy was not only ineffective but also might be associated with harm in certain subgroups [70].

Current Controversies and Future Research

These mixed results have raised several concerns. First, vitamin C may not benefit all septic patients equally. Differences in timing of administration, severity of illness, and baseline vitamin C status could influence outcomes. Additionally, the optimal dose, duration, and whether to use it as monotherapy or in combination remain unresolved questions [71]. Ongoing trials are attempting to refine these variables, but for now, the role of vitamin C in routine sepsis care remains uncertain. Its use should be considered experimental and ideally reserved for clinical trials or certain patient groups under careful supervision.

Role of corticosteroids in sepsis management

Mechanisms of Action

There is a strong rationale for using corticosteroids to treat sepsis; nonetheless, their application in clinical settings continues to be a subject of debate. Excessive systemic inflammation acts as a primary cause of severe sepsis and the sequelae of organ dysfunction and death [72]. Anti-inflammatory properties are induced by glucocorticoids through the synthesis of anti-inflammatory proteins and the subsequent inhibition of pro-inflammatory proteins. Glucocorticoids bind to a specific intracellular receptor encoded on chromosome 5q31-31 [73]. On binding with glucocorticoids, the glucocorticoid-receptor complex interacts with specific deoxyribonucleic acid (DNA) sequences. Chromatin remodeling is involved in the modulation of genetic expression [74,75]. Activated receptors reduce the expression of pro-inflammatory genes by inhibiting histone acetyltransferases and promoting the activity of histone deacetylases. The receptor-glucocorticoid complex also prevents the activity of pro-inflammatory proteins by sequestering transcription factors like NF-kB, which is involved in the production of pro-inflammatory cytokines [76,77]. Glucocorticoids also counter inflammatory responses by inducing the production of annexin 1 that inhibits the expression of phospholipase A2. Phospholipase A2 catabolizes the production of arachidonic acid elements such as prostaglandins and leukotrienes, which are involved in pain and inflammatory pathways [78]. During sepsis, several chemokines and inflammatory cytokines are involved including IL-1B, IL-6, IL-12, and IL-7 [24]. The relationship between sepsis and cytokine storm has been considered to be essential in the discovery of effective therapeutic interventions [24]. There is evidence that highlights the usage of corticosteroids in patients diagnosed with refractory septic shock. In such patients, moderate dosage corticosteroids might play a role in restoring sensitivity of cells to vasopressors and improving hemodynamic stability [79,80]. This decreases the need for vasopressors and is mediated by vascular tone independent of adrenal function tests [81]. Corticosteroids also contribute to the restoration of blood volume via sodium and water retention through mineralocorticoid activity, and help to improve systemic vascular resistance by enhancing vascular contractile and blood pressure responses to alpha-1 agonists [81,82].

Key Clinical Trials

The traditional use of corticosteroids such as methylprednisolone in sepsis management posed no significant reduction in mortality and presented with adverse effects [83,84]. This led to experimentation on new treatment modalities involving other corticosteroids such as hydrocortisone using prolonged low doses [85]. Multiple small-scale studies found that administering 200-400 mg of hydrocortisone daily for more than three days enhanced cardiovascular function in patients with sepsis [86]. A prolonged treatment with low to moderate doses of corticosteroids in septic shock, along with evidence of adrenal insufficiency, showed an enhancement in survival rates and cardiovascular functions in patients experiencing septic shock [80]. In accordance with the GERINF05 trial, the Activated Protein C and Corticosteroids for Human Septic Shock (APROCCHSS) trial found that the combination of hydrocortisone and fludrocortisone at low doses resulted in a decrease in 90-day mortality for patients experiencing septic shock [80,87]. The Corticosteroid Therapy of Septic Shock (CORTICUS) trial failed to replicate the survival benefits observed in GERINF05, while still affirming its effects on cardiovascular function [81]. In the Adjunctive Corticosteroid Treatment in Critically Ill Patients With Septic Shock (ADRENAL) trial, which focused on critically ill patients with septic shock, a continuous infusion of hydrocortisone in patients on mechanical ventilation did not lead to a significant decrease in mortality when compared to those receiving a placebo [87,88].

Current Recommendations

The uncertainty about the potency of corticosteroids in the management of sepsis has led to varied implementation in clinical practice [88]. In view of these, the guidelines from the 2016 Surviving Sepsis Campaign (SSC) recommend administering glucocorticoids for patients experiencing septic shock when sufficient fluid resuscitation and vasopressor treatment do not achieve hemodynamic stability [89,90].

Role of thiamine in sepsis management

Mechanisms of Action

The supplementation of thiamine in the management of sepsis may be justified by its protective effect on mitochondrial activity, as mitochondrial dysfunction contributes to the pathophysiology of the syndrome [90]. This may then result in oxidative phosphorylation impairment aided by inflammatory signaling pathways [8]. Thiamine serves as a metabolic resuscitator, restoring mitochondrial function and aiding in energy metabolism. The active form of thiamine, known as thiamine pyrophosphate, serves as a cofactor for the pyruvate dehydrogenase complex and is crucial for the transformation of pyruvate into acetyl coenzyme A, leading to oxidative phosphorylation and the production of ATP [91]. The deficiency of thiamine impairs pyruvate dehydrogenase function and causes pyruvate to be converted into lactic acid. This would result in an energy deficit and lactic acidosis [92]. Thiamine pyrophosphate is also a cofactor for alpha ketoglutarate dehydrogenase, a mitochondrial enzyme that converts alpha ketoglutarate to succinyl coA [93]. Energy production and the creation of reactive oxygen species account for over 95% of these metabolic functions [94]. Thiamine also plays a role in antioxidant pathways, including glutathione cycling, which aim to restore metabolic function in patients experiencing septic shock. It also provides resistance to the oxidative stress of cells, which usually accompanies sepsis via the regulation of glucose metabolism [95,96]. The supplementation of both thiamine and vitamin C has been linked to restore organ function during sepsis and septic shock [97]. This is made possible by the ability of thiamine to reduce renal oxalate crystallization by preventing the conversion of vitamin C to oxalate, which can be harmful [98].

Key Clinical Studies

In a large-sized study, it was demonstrated that intravenous thiamine led to lower mortality rates and enhanced lactate clearance [99]. Only two randomized clinical trials involving thiamine as a standalone nutrient have been published. In the first trial, patients with septic shock received 200 mg of intravenous thiamine or a placebo for days or until hospital discharge. Supplementation was found to not be effective in reducing serum lactate after the first 24 hours [100]. A secondary analysis of this trial indicated that the group receiving treatment had lower serum creatinine levels and a reduced need for renal replacement therapy compared to the placebo group [95]. There was no difference in mortality; however, it suggests the potential significance of thiamine as a renal protective agent in cases of septic shock. Another randomized trial assessed vasopressor-free days in septic shock patients who received 20 mg of intravenous thiamine or placebo for several days or until the patients were discharged from the hospital. Thiamine supplementation did not show a decrease in the requirement for vasoactive medications during the initial week of intensive care unit admission or impact 28-day mortality. Conversely, the study revealed noteworthy findings, including a decrease in the vasopressor dependency index and in the serum lactate levels 24 hours following the initial supplementation [34]. In another clinical study designed to test the combined use of thiamine with vitamin C and corticosteroids, patients experiencing severe sepsis or septic shock received treatment through the administration of a combination of vitamin C, dosed at 1.5 g every 6 hours, hydrocortisone (50 mg every 6 hours) and thiamine (200 mg every 12 hours) for a duration of four days. During the control period, patients diagnosed with sepsis were not administered intravenous vitamin C or thiamine. The treated group experienced a mortality rate that was significantly lower than that of the control group [101].

Current Use and Controversies

In clinical practice, measuring plasma thiamine levels is not a commonly available laboratory test and can take several days to complete, making it unhelpful for decision-making in cases of septic shock. The guidelines for parenteral nutrition in intensive care from the European Society for Clinical Nutrition and Metabolism advised administering thiamine supplementation at a dosage of 100 to 300 mg per day for the initial three days in the intensive care unit for all patients suspected of having thiamine deficiency [102]. Research indicates that administering thiamine may be advantageous not only for septic patients with a deficiency but also for those at risk of thiamine deficiency, given that intravenous thiamine is affordable and has minimal side effects. Factors that contribute to thiamine deficiency include malnutrition, alcoholism, chronic wasting diseases, renal replacement therapy, gastric bypass surgery, and refeeding [102].

Vitamin C, steroids, and thiamine (HAT) protocol: synergy and challenges

Theoretical Synergy of the Three Components

Recently, there has been a proposal to use a combination of steroids, vitamin C, and thiamine alongside conventional treatments for sepsis as a form of triple therapy. In comparison to each drug being used individually, the combined effects of all three agents seem to significantly influence the progression of sepsis, enhancing outcomes [4]. Thiamine in conjunction with vitamin C suppresses oxidative stress and inhibits oxalate conversion, thereby prolonging its effects [98]. With the addition of hydrocortisone, vitamin C inhibits the activation of nuclear factor signaling pathways and downregulates the synthesis of pro-inflammatory mediators. Steroids, mainly hydrocortisone, also enhance the production of vitamin C by facilitating the expression of the ascorbate transporter protein (sodium-dependent vitamin C transporter 2, or SVCT2), while vitamin C has the potential to decrease the oxidation of the hydrocortisone receptor and improve its efficacy. At the same time, hydrocortisone combined with vitamin C synergistically prevents and repairs lipopolysaccharide-induced damage to human pulmonary microvascular endothelial cells and reverses septic shock compared with hydrocortisone or vitamin C alone [68]. Reportedly, the use of vitamin C and thiamine in patients with sepsis and septic shock also led to a decrease in the duration of vasopressor administration [103].

Key Clinical Trials on Combination Therapy

Numerous registered clinical trials have investigated this combination therapy, yielding varied results. A randomized clinical trial explored the efficacy of hydrocortisone, ascorbic acid, thiamine therapy in patients with sepsis and found that the prompt initiation of HAT therapy significantly reduced the duration of vasopressin administration. However, the study was unable to determine the relationship between HAT therapy and improvement in hospital and intensive care mortality in patients with septic shock. The study did not identify any significant adverse events linked to the drugs, indicating that the therapy is comparatively safe. Several other clinical trials reported similar results of reducing the duration of vasopressors in septic patients with no significant reduction in mortality, when using HAT therapy [104-107]. Deviating from the other studies, a trial in China discovered that this combination therapy did not reduce the duration of vasopressor use in patients, but it did lead to a significant improvement in Sequential Organ Failure Assessment (SOFA) scores at the 72-hour mark [108]. Significant benefits of HAT therapy could not be ascertained from several clinical trials. A study indicated that it had no effect on the duration of ventilator-free days or the use of vasopressors within a 30-day timeframe, nor on the 180-day mortality rate in patients suffering from septic shock [109].

Current Guidelines and Expert Opinions

The international guidelines for the management of sepsis and septic shock, by the SSC, offer recommendations for the treatment of patients with sepsis or those who are at risk [110]. Contrary to the 2016 guidelines that advised against administering intravenous hydrocortisone for treating septic shock patients when fluid resuscitation or vasopressors were sufficient to stabilize hemodynamics, the 2021 guidelines now recommend the use of intravenous corticosteroids for adults experiencing septic shock who continue to require vasopressors [2]. This revised recommendation factors in several randomized clinical trials where corticosteroid therapy was shown to reduce the duration of shock, but its effect on mortality remains uncertain [80,87]. The recommendation is weak and suggests the use of intravenous corticosteroids for adult patients experiencing septic shock who continue to need vasopressor therapy. If the patient no longer needs vasopressor therapy or is no longer in shock, and is sustaining adequate blood pressure for a minimum of 12 to 24 hours, the new guidelines recommend tapering or stopping steroids. Steroid use should be guided by clinical response and assessed on a case basis. The 2021 guidelines also discourage the use of intravenous vitamin C for septic shock, drawing on findings from meta-analyses of randomized clinical trials, such as the recent LOVIT trial [70], rather than observational data [2,111].

Challenges and future directions

Identifying the Right Patient Population

The diagnosis and treatment of sepsis present a challenge primarily due to the non-specific nature of the symptoms. Traditional methods lack a definitive standard and can be time consuming. Recent developments indicate potential in improving sepsis management by utilizing biomarker-driven strategies. Incorporating biomarkers into sepsis management protocols could enhance the early diagnosis of sepsis and inform antibiotic stewardship initiatives. This will assist in risk stratification and monitor treatment response that will help inform clinical decision-making [69,112]. Certain developed biomarkers like procalcitonin and C-reactive protein continue to be valuable indicators of infection and inflammation. This helps to monitor treatment response and guide antibiotic therapy. The utilization of biomarkers also creates room for a more individualized approach that combines rapid recognition, early intervention, and targeted therapy based on patient characteristics and response to therapy. This shift has been reflected in recent guidelines including the 2021 SSC recommendations [113]. Further novel clinical research is required to investigate how the incorporation of biomarkers into treatment protocols may enhance patient outcomes in cases of sepsis or septic shock.

Optimal Dosage and Administration Strategies

The optimal dosage and administration strategies for vitamin C, corticosteroids and thiamine vary. Most clinical trials adopt the fixed-dose strategy with 1.5 g of intravenous ascorbic acid every six hours for four days [1,101,108]. Other trials, including the recent LOVIT trial, have used weight-based dosing of vitamin C at 50 mg/kg [114]. This vitamin C dosage regimen may not be the most effective for preventing the pathophysiological processes involved in sepsis. Thiamine supplementation has not been linked to any serious adverse effects, even when taken in high doses [115]. Up to 500 mg per dose (IV) may be necessary for patients experiencing septic shock. Thiamine should be given intravenously over a period of 15 to 30 minutes in a mixture of saline solution or dextrose to prevent possible adverse reactions [116]. Hydrocortisone is usually preferred to synthetic corticosteroids. The commonly accepted dosage is 200 mg per day on average. A recent meta-analysis revealed that a lower dose of corticosteroids is associated with a stronger response to treatment [117]. Hydrocortisone may be given as boluses or as a continuous infusion.

Large-Scale RCTs

Research based on evidence has indicated that administering vitamin C can have a beneficial effect on patients with severe sepsis and septic shock. Preliminary studies have indicated a beneficial effect on multiple-organ failure and endothelial injury [118]. In the Vitamin C Therapy or Routine Care in Septic Shock (ViCTOR) trial, the HAT protocol did not reduce in-hospital mortality in patients with septic shock within six hours of diagnosis [107]. In critically ill patients, endogenous antioxidants such as glutathione peroxidase and reduced glutathione have been used as indicators of antioxidative status and disease severity. While these initial findings are promising, the mixed outcomes observed across studies highlight the need for large-scale randomized controlled trials to provide definitive evidence on the efficacy and safety of vitamin C in sepsis management. Such trials are crucial to reconcile current inconsistencies, validate potential benefits, and inform clinical guidelines with confidence.

## Conclusions

Vitamin C, corticosteroids, and thiamine represent a theoretically promising yet clinically debated strategy in sepsis management. While corticosteroids are currently recommended for patients with septic shock, vitamin C and thiamine remain investigational according to Surviving Sepsis Campaign guidelines. Moving forward, precision medicine approaches that incorporate biomarker-driven patient selection may help identify individuals most likely to benefit from metabolic resuscitation. High-quality randomized clinical trials and translational research are essential to refine therapeutic strategies and strike an optimal balance between standardized protocols and individualized care before widespread adoption.
